# Caffeine Targets G6PDH to Disrupt Redox Homeostasis and Inhibit Renal Cell Carcinoma Proliferation

**DOI:** 10.3389/fcell.2020.556162

**Published:** 2020-10-06

**Authors:** Huanhuan Xu, Lihong Hu, Titi Liu, Fei Chen, Jin Li, Jing Xu, Li Jiang, Zemin Xiang, Xuanjun Wang, Jun Sheng

**Affiliations:** ^1^Key Laboratory of Pu-er Tea Science, Ministry of Education, Yunnan Agricultural University, Kunming, China; ^2^College of Science, Yunnan Agricultural University, Kunming, China; ^3^College of Food Science and Technology, Yunnan Agricultural University, Kunming, China; ^4^State Key Laboratory for Conservation and Utilization of Bio-Resources in Yunnan, Kunming, China

**Keywords:** caffeine, G6PDH activity, redox homeostasis, renal cell carcinoma, tumor growth

## Abstract

Glucose-6-phosphate dehydrogenase (G6PDH) is the rate-limiting enzyme in the pentose phosphate pathway (PPP) and plays a crucial role in the maintenance of redox homeostasis by producing nicotinamide adenine dinucleotide phosphate (NADPH), the major intracellular reductant. G6PDH has been shown to be a biomarker and potential therapeutic target for renal cell carcinoma (RCC). Here, we report a previously unknown biochemical mechanism through which caffeine, a well-known natural small molecule, regulates G6PDH activity to disrupt cellular redox homeostasis and suppress RCC development and progression. We found that caffeine can inhibit G6PDH enzymatic activity. Mechanistically, caffeine directly binds to G6PDH with high affinity (*K*_D_ = 0.1923 μM) and competes with the coenzyme NADP^+^ for G6PDH binding, as demonstrated by the decreased binding affinities of G6PDH for its coenzyme and substrate. Molecular docking studies revealed that caffeine binds to G6PDH at the structural NADP^+^ binding site, and chemical cross-linking analysis demonstrated that caffeine inhibits the formation of dimeric G6PDH. G6PDH inhibition abrogated the inhibitory effects of caffeine on RCC cell growth. Moreover, inhibition of G6PDH activity by caffeine led to a reduction in the intracellular levels of NADPH and reactive oxygen species (ROS), and altered the expression of redox-related proteins in RCC cells. Accordingly, caffeine could inhibit tumor growth through inhibition of G6PDH activity *in vivo*. Taken together, these results demonstrated that caffeine can target G6PDH to disrupt redox homeostasis and inhibit RCC tumor growth, and has potential as a therapeutic agent for the treatment of RCC.

## Introduction

Redox reactions represent a predominant and dynamic metabolic process that maintains human health (Ray et al., 2012). An imbalance of redox homeostasis can result in numerous diseases, including cancer (Ju et al., 2017; Liu et al., 2019). A sustained increase in the production of ROS has been detected in most cancers, promoting both tumorigenesis and tumor progression (Waris and Ahsan, 2006; Liou and Storz, 2010; Ciccarese et al., 2020). However, tumor cells also can detoxify from ROS by upregulating the expression levels of antioxidant enzymes, indicating that disturbance of a delicate balance in intracellular ROS levels contributes to cancer cell function (Liou and Storz, 2010).

Nicotinamide adenine dinucleotide phosphate (NADPH) is the main intracellular reductant, and plays critical roles in reductive biosynthesis and the maintenance of redox homeostasis in cells, as well as in cellular processes regulated by redox signaling pathways (Yang et al., 2019). Cytosolic glucose-6-phosphate dehydrogenase (G6PDH) catalyzes the first and rate-limiting step of the oxidative branch in the PPP. The PPP represents an alternative route for glycolysis for the dissimilation of carbohydrates, and is a major source of reducing power and metabolic intermediates for fatty acid and nucleic acid biosynthetic processes (Kotaka et al., 2005; Jiang et al., 2014). These biomasses produced through the PPP have an important role in cancer cell growth (Jiang et al., 2014). Studies have shown that elevated G6PDH activity is frequently observed in many human cancers, and is a predictor for poor prognosis in cancer patients, suggesting that G6PDH plays crucial roles in cancer development and progression (Riganti et al., 2012; Patra and Hay, 2014). Emerging evidence from *in vitro* and *in vivo* studies has demonstrated that inhibition of the rate-limiting enzyme G6PDH in PPP can strongly suppress tumor cell growth (Mele et al., 2018), suggesting that G6PDH may be a potential therapeutic target for exploring effective cancer treatment modalities (Pandolfi et al., 1995; Zhang et al., 2014).

Renal cell carcinoma is the most prevalent and dangerous renal malignancy. RCC accounts for approximately 3% of all tumor types, and its incidence continues to rise. The median survival time for RCC patients is only approximately 13 months, and fewer than 10% will survive more than 5 years (Busch et al., 2015; Zhang et al., 2017b). It has been estimated that approximately 73,750 new cases of kidney and renal pelvis cancers will be diagnosed and 14,830 people will die from this condition in the United States in 2020 (Siegel et al., 2020). Considerable accumulated evidence has demonstrated that G6PDH exhibits higher enzyme activities in RCC patients, which could be used as a biomarker for clinical diagnosis (Spencer and Stanton, 2017; Zhang et al., 2017b). Caffeine (1,3,7-trimethylxanthine), a naturally occurring plant xanthine alkaloid found in tea, coffee, cocoa, and many other food products, has been shown to possess numerous biological functions (Xu et al., 2019). A previous case–control study concluded that caffeine consumption can reduce the risk of developing RCC (Antwi et al., 2017). However, whether caffeine can affect RCC development and progression remains unknown.

Given that caffeine shares a similar purine structure with the coenzyme NADP^+^ and the important roles of G6PDH-mediated redox homeostasis in RCC development and progression, we hypothesized that caffeine might compete with NADP^+^ for binding to G6PDH, thereby disrupting redox homeostasis and suppressing RCC proliferation. In this study, we showed that caffeine can directly bind to G6PDH with high affinity and compete with NADP^+^ for G6PDH binding, which inhibits G6PDH activity, disrupts redox homeostasis, and leads to suppression of RCC proliferation, both *in vitro* and *in vivo*. These findings suggest that caffeine can target G6PDH to disrupt redox homeostasis and inhibit RCC tumor growth, and has potential for use as a therapeutic agent in the treatment of RCC.

## Materials and Methods

### Chemicals and Reagents

High-purity grade (≥98%) caffeine and crystal violet were purchased from Aladdin Bio-Chem Technology, Co., Ltd. (Shanghai, China). NADP, 3-(4,5-dimethylthiazol-2-yl)-2,5-diphenyltetrazolium bromide (MTT), and a NADP/NADPH Quantification Kit were obtained from Sigma-Aldrich (St. Louis, MO, United States). D-Glucose 6-phosphate, recombinant G6PDH protein, and the G6PDH Activity Colorimetric Assay Kit were purchased from BioVision Incorporated (Milpitas, CA, United States). Reactive Oxygen Species Detection Reagents and DMEM were purchased from Thermo Fisher Biochemical Products (Beijing) Co., Ltd. FBS and a mixed P/S were purchased from Biological Industries (Kibbutz Beit Haemek, Israel) and Solarbio (Beijing, China), respectively. Primary antibodies against G6PDH, NOX4, NOX2, SOD2, and Cyclin D1 were obtained from Abcam (Cambridge, MA, United States). Antibodies against STAT3, p-STAT3, Ki67, and β-actin were purchased from Cell Signaling Technology (Beverly, MA, United States). Anti-Catalase, anti-p47-phox, and anti-Cyclin E antibodies were purchased from Santa Cruz Biotechnology (Santa Cruz, CA, United States). Anti-β-tubulin and horseradish peroxidase-conjugated secondary antibodies were purchased from Sino Biological, Inc. (Beijing, China) and R&D Systems (Minneapolis, MN, United States), respectively.

### Surface Plasmon Resonance (SPR) Analysis

Surface plasmon resonance studies were carried out using a Biacore S200 instrument (GE Healthcare, Uppsala, Sweden) at 25°C. G6PDH protein was immobilized on the Series S CM5 Sensor Chip using the standard amine-coupling method. G6PDH protein was diluted to 50 μg/mL in 10 mM sodium acetate buffer, pH 4.5. Immobilization was performed using an amine-coupling kit (GE Healthcare) following the manufacturer’s protocol. The kinetics and affinity assay were determined at a flow rate of 30 μL/min using PBS-P buffer [20 mM phosphate buffer, 2.7 mM KCl, 137 mM NaCl, and 0.05% (*v/v*) P20 surfactant]. Diluted caffeine, NADP^+^, and G6P were stored at 4°C and placed into the rack tray before injection. The association and dissociation times were both 90 s. The *K*_D_ values were calculated with the kinetics and affinity analysis option of Biacore S200 Evaluation Software Version 1.1 (GE Healthcare).

### Molecular Docking Studies

The X-ray crystal structure of G6PDH (PDB code: 2BH9) was retrieved from the Protein Data Bank^1^. MVD software v2011.5.0 was used to perform the molecular docking studies in accordance with the literature (Benatti et al., 1978; Velasco-Garcia et al., 2000; Kotaka et al., 2005). Docking parameters were set to default values according to the Docking Wizard program. The lowest binding energy model from the 10 candidate conformers was selected for each docking simulation and the resultant data were analyzed. The model of the caffeine-G6PDH-NADP^+^ complex was further displayed.

### Chemical Cross-Linking Analysis

The G6PDH protein was treated with 0.025% glutaraldehyde in the absence or presence of caffeine (80 μM). The formation of G6PDH monomer and dimer was determined by Coomassie blue staining and a FluorChem E System (ProteinSimple, San Jose, CA, United States).

### Cell Lines and Cell Culture

The human RCC cell lines ACHN and 786-O were purchased from the American Type Culture Collection (ATCC; Manassas, VA, United States). The cells were cultured in DMEM supplemented with 10% FBS and 1% P/S at 37°C in a humidified incubator with 5% CO_2_ (BINDER GmbH, Tuttlingen, Germany).

### Cell Viability Assay

The effect of caffeine on the viability of ACHN and 786-O cells was examined using the standard MTT method as previously described (Liu et al., 2017). Briefly, ACHN and 786-O cells were seeded into 96-well plates at a density of 1.5 × 10^4^ cells/well and then treated with various concentrations of caffeine (0–3200 μg/mL) for 48 h. After incubating the cells with 20 μL of MTT solution (5 mg/mL) for 4 h, the supernatant was aspirated and 200 μL of DMSO was added to dissolve the formazan crystals. Absorbance was read at 492 nm using a FlexStation 3 Multi-Mode Microplate Reader (Molecular Devices, Sunnyvale, CA, United States), and IC_50_ values were further calculated.

### Colony Formation Assay

Viable ACHN and 786-O cells (4 × 10^3^ cells/plate) were seeded into 60-mm plates. After adhering overnight, the indicated agents were added to the cells, with PBS being used as a control. The culture medium was changed every 2 days. After incubation for 1 week, the cells were fixed in 4% paraformaldehyde and then stained with crystal violet. After multiple washes, the plates were air-dried and imaged, and individual clones were scored.

### Cell Apoptosis Assay

Cell apoptosis was detected using an Annexin V-FITC/Propidium Iodide (PI) kit (US Everbright^®^ Inc., Suzhou, China) according to the manufacturer’s instructions. ACHN and 786-O cells were treated with or without caffeine. After 24 or 48 h, the cells were collected and incubated in 200 μL of 1 × binding buffer and 20 μL of Annexin V-FITC, followed by staining with 20 μL of PI for 15 min at room temperature in the dark. Subsequently, the cells were subjected to flow cytometry (BD FACSCalibur, Wayne, PA, United States) analysis.

### G6PDH Activity Assay

The effect of caffeine on G6PDH activity was determined using a G6PDH Activity Colorimetric Assay Kit (BioVision). All cells were seeded at 6 × 10^5^ cells per 60-mm plate and treated either with PBS (Control) or caffeine. After 24 or 48 h, the cells were lysed and quantified, a NADH standard curve was generated, and equal amounts of proteins were determined at 450 nm in the kinetic model for 30 min at 37°C in the dark according to the manufacturer’s instructions. In addition, the effect of caffeine on G6PDH enzymatic activity and G6PDH activity in xenograft tumor tissues were both measured.

### Western Blotting Analysis

Proteins were extracted in RIPA lysis buffer (Solarbio) containing 1 mM PMSF (Solarbio). Samples containing equal amounts of protein were separated by SDS–PAGE and electrophoretically transferred onto polyvinylidene fluoride (PVDF) membranes (Millipore; Merck KGaA, Darmstadt, Germany). The membranes were probed with primary antibodies overnight at 4°C, and then incubated with the corresponding anti-rabbit or anti-mouse secondary antibodies conjugated to horseradish peroxidase for 1 h at room temperature. The bands of interest were detected using an Ultra-sensitive Enhanced Chemiluminescent Substrate Kit (4A Biotech, Co. Ltd., Beijing, China) and a FluorChem E System (ProteinSimple), and were further quantified using AlphaView software (Cell Biosciences, Santa Clara, CA, United States).

### Inhibition of G6PDH by 6-Aminonicotinamide or Palmitate

ACHN and 786-O cells (1.5 × 10^4^ cells/well) were seeded into 96-well plates and incubated overnight. The cells were pretreated with or without 6-Aminonicotinamide (6-AN, 20 μM) or palmitate (PA, 500 μM) for 4 h and subsequently stimulated with caffeine for 48 or 12 h. After that, the cells were used for cell viability assay.

### Assays for NADPH and ROS Levels

NADPH levels were detected using a NADP/NADPH Quantification Kit. Viable ACHN and 786-O cells (4 × 10^5^ cells/plate) were seeded into 60-mm plates and incubated overnight. After treatment with caffeine for 24 or 48 h, the cells were lysed, and samples were prepared for detecting NADPH levels. A NADPH standard curve was generated, and NADPH in the samples was measured at 450 nm according to the manufacturer’s instructions. Additionally, intracellular ROS levels were determined with CM-H_2_DCFDA (Baek et al., 2017). ACHN and 786-O cells (1 × 10^5^ cells/well) were seeded into 12-well plates and allowed to adhere overnight. After treatment with caffeine for 24 or 48 h, the cells were incubated in phenol red-free medium containing 10 μM CM-H_2_DCFDA for 25 min in the dark. Images were captured at × 200 magnification under a fluorescence microscope (Leica Microsystems, Wetzlar, Germany) and further analyzed using ImageJ software (National Institutes of Health, Bethesda, MD, United States). In addition, the levels of ROS were measured with a flow cytometer (BD FACSCalibur).

### *In vivo* Xenograft Studies

All animal experiments were carried out in accordance with the Yunnan Agricultural University Animal Care Facility and National Institutes of Health guidelines. Fifteen female BALB/c nude mice were sourced from Cawens Lab Animal, Co. (Changzhou, China). After acclimation for 1 week, ACHN (5 × 10^6^) and 786-O (4.5 × 10^6^) cells were suspended in 150 μL of DMEM and injected subcutaneously into the left and right flanks of each mouse, respectively. Tumor volume was examined by Vernier caliper and calculated based on the following formula: width × width × length/2 every 2 days. When tumor size had reached approximately 50 mm^3^, the tumor-bearing mice were randomly allocated to three groups. Caffeine was administered continuously for 34 days to low- and high-dose caffeine groups at 60 and 120 mg/kg body weight/day, respectively, *via* the intragastric route; the control group was intragastrically administered the same volume of sterile water. After the treatment period, the mice were euthanized by deep ether anesthesia and the isolated tumors were weighed, photographed, and further used for detection of G6PDH activity, immunohistochemical staining, and western blot analysis.

### Immunohistochemical Staining

Formalin-fixed, paraffin-embedded xenograft tumor tissues were cut into 3 μm-thick sections for immunohistochemistry. After antigen retrieval, slides were incubated with primary antibodies overnight at 4°C. Immunostaining was performed using the VECTASTAIN Elite ABC-Peroxidase Kit (Vector Laboratories, Burlingame, CA, United States) and an Enhanced HRP–DAB Chromogenic Kit (TIANGEN Biotech, Co., Ltd., Beijing, China) according to the manufacturer’s instructions. The slides were then counterstained with Mayer’s hematoxylin (Sigma-Aldrich), dehydrated, and cover-slipped using mounting solution. Images were captured at × 400 magnification under a CKX41 microscope (Olympus, Tokyo, Japan) and further analyzed using Image-Pro Plus 6.0 software (Media Cybernetics, Inc., Rockville, MD, United States).

### Statistical Analysis

All results are presented as means ± standard error of the mean (SEM) of three or more independent replicates. The Student’s *t*-test was applied to determine significance using GraphPad Prism 5.0 software (GraphPad Software, Inc., La Jolla, CA, United States). *P* < 0.05 was considered significant. Representative images are displayed.

## Results

### Caffeine Inhibits G6PDH-Mediated Enzymatic Activity *in vitro*

**FIGURE 1:**
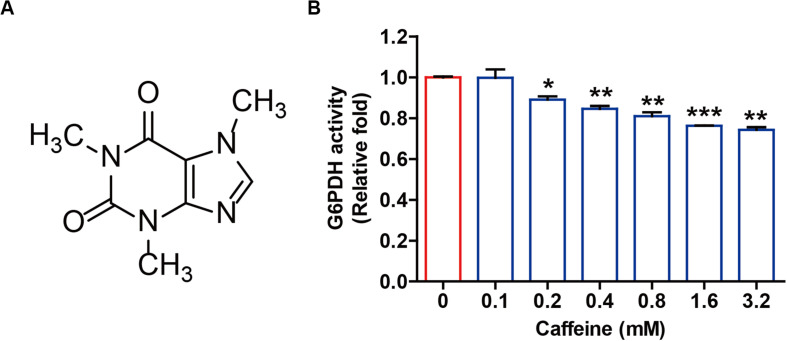
Caffeine directly inhibits G6PDH activity in a pure enzyme reaction system. **(A)** The chemical structure of caffeine. **(B)** Caffeine inhibits G6PDH activity in an *in vitro* system. The effect of caffeine on G6PDH enzymatic activity was determined using a G6PDH Activity Colorimetric Assay Kit. *P < 0.05, **P < 0.01, and ***P < 0.001 versus the control group. Data are shown as means ± SEM of triplicated experiments.

Caffeine is a highly chemically stable xanthine alkaloid with three methyl groups at the 1, 3, and 7 positions of the purine ring structure (Figure 1A), and not readily oxidized under normal physiological conditions. Given the structural similarity between caffeine and NADP^+^ and the important roles of NADP^+^ as a coenzyme for G6PDH enzymatic activity, we directly investigated whether caffeine influenced G6PDH-mediated enzymatic activity *in vitro*. As shown in Figure 1B, caffeine could directly inhibit G6PDH activity in a pure enzyme reaction system in a concentration-dependent manner. This result suggested that caffeine could directly inhibit G6PDH enzymatic activity and might directly interact with G6PDH.

### Caffeine Directly Interacts With G6PDH and Decreases Its Coenzyme and Substrate Binding Affinities

**FIGURE 2:**
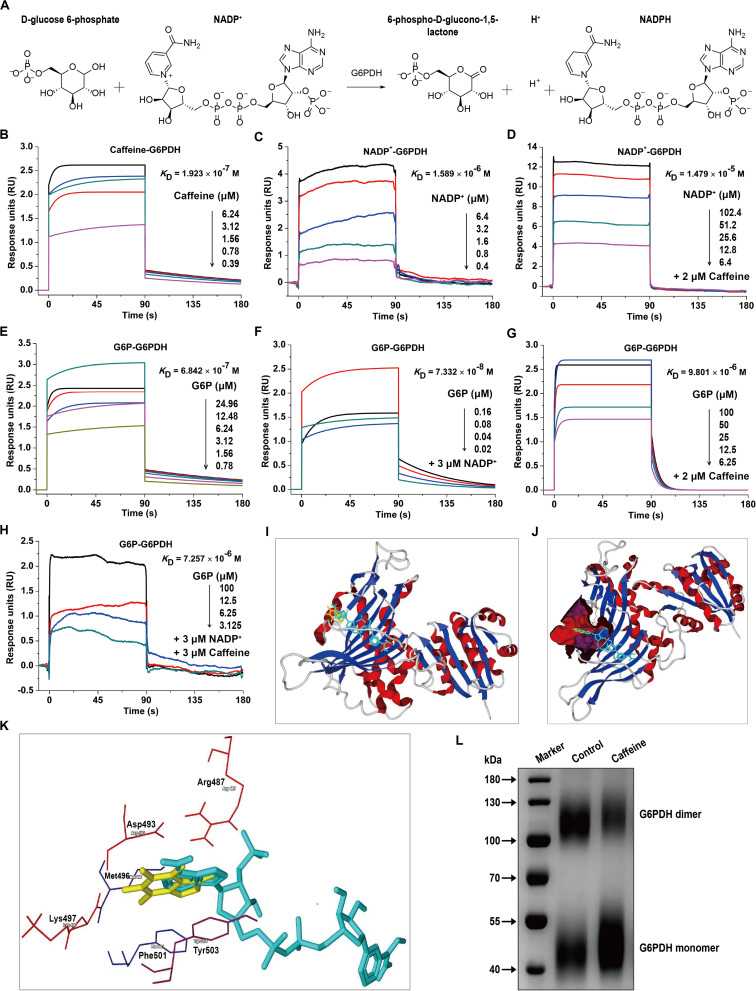
Caffeine directly binds to G6PDH and decreases its coenzyme and substrate binding affinities. **(A)** The enzymatic reaction process involving G6PDH. **(B)** Caffeine directly binds to G6PDH, with a binding affinity of *K*_D_ = 1.923 × 10^– 7^ M. **(C)** The coenzyme NADP^+^ binds to G6PDH, with *K*_D_ = 1.589 × 10^– 6^ M. **(D)** NADP^+^ binds to G6PDH in the presence of caffeine (2 μM), with *K*_D_ = 1.479 × 10^– 5^ M. **(E)** The substrate G6P binds to G6PDH, with *K*_D_ = 6.842 × 10^– 7^ M. **(F)** G6P binds to G6PDH in the presence of NADP^+^ (3 μM), with *K*_D_ = 7.332 × 10^– 8^ M. **(G)** G6P binds to G6PDH in the presence of caffeine (2 μM), with *K*_D_ = 9.801 × 10^– 6^ M. **(H)** G6P binds to G6PDH in the presence of NADP^+^ (3 μM) and caffeine (3 μM), with *K*_D_ = 7.257 × 10^– 6^ M. **(I–K)** The computational binding mode of caffeine to human G6PDH (PDB code: 2BH9) was established using the MVD molecular docking software. **(I)** Caffeine (yellow) binds to G6PDH (ribbon) at the structural NADP^+^ (light blue) binding site. **(J)** Caffeine (yellow) and NADP^+^ (light blue) are both bound in the same pocket (magenta) of G6PDH (ribbon). **(K)** The binding pocket for caffeine (yellow) and NADP^+^ (light blue) is formed by six important residues in G6PDH, shown in sticks: Arg487, Asp493, Met496, Lys497, Phe501, and Tyr503. **(L)** Caffeine inhibits the formation of dimeric G6PDH, as demonstrated by chemical cross-linking analysis. All SPR experiments were performed using a Biacore S200 instrument. The data displayed here represent one of three independent experiments with similar results.

G6PDH is the rate-limiting enzyme in the PPP and plays crucial roles in NADPH generation (Wang et al., 2014). G6PDH catalyzes the oxidation of D-glucose-6-phosphate (G6P) to 6-phospho-D-glucono-1,5-lactone and concomitantly reduces NADP^+^ to NADPH (Figure 2A). To investigate whether caffeine directly binds to G6PDH and elucidate the molecular mechanisms underlying the caffeine-mediated inhibition of G6PDH activity, we performed SPR studies using a Biacore S200 instrument, which is widely applied to determine kinetic and affinity constants for small molecule compound–protein interactions in real-time without labels (Wang et al., 2016).

Interestingly, we found that caffeine could directly bind to G6PDH with an equilibrium dissociation constant (*K*_D_) of 0.1923 μM (Figure 2B), indicating that the caffeine-G6PDH complex is very stable. As shown in Figure 2C, G6PDH bound to its coenzyme NADP^+^ with a *K*_D_ of 1.589 μM, similar to the result reported in a previous study (Kotaka et al., 2005). However, the addition of caffeine (2 μM) inhibited the interaction between G6PDH and NADP^+^, with the *K*_D_ value decreasing to 14.79 μM (Figure 2D), suggesting that caffeine could compete with NADP^+^ for G6PDH binding. In addition, G6PDH bound to its substrate G6P with a *K*_D_ of 0.6842 μM (Figure 2E); as expected, the presence of the coenzyme NADP^+^ (3 μM) enhanced the binding affinity of G6PDH for G6P, with a *K*_D_ of 0.07332 μM (Figure 2F). However, caffeine (2 μM) also inhibited the interaction between G6PDH and G6P, with the *K*_D_ value decreasing to 9.801 μM (Figure 2G). Importantly, when caffeine (3 μM) was added to the complete G6PDH reaction system, the binding affinity of G6PDH to G6P was decreased from 0.07332 to 7.257 μM (Figure 2H). Taken together, these results demonstrated that caffeine directly binds to G6PDH with high affinity and decreases its coenzyme and substrate binding affinities.

To better understand the interaction between caffeine and G6PDH, the possible caffeine-G6PDH (PDB code: 2BH9) binding models were evaluated using the MVD molecular docking software. Figure 2I illustrates the most probable caffeine-G6PDH-NADP^+^ binding model. Interestingly, we found that caffeine bound to G6PDH at the structural NADP^+^ binding site (Figure 2I), further confirming that caffeine can compete with the coenzyme NADP^+^ for G6PDH binding. In addition, caffeine and NADP^+^ both bound the same pocket of G6PDH (Figure 2J), which was formed by six important residues in G6PDH: Arg487, Asp493, Met496, Lys497, Phe501, and Tyr503 (Figure 2K). Notably, hydrophobic interaction forces play a major role in the interaction between caffeine and G6PDH. More importantly, chemical cross-linking analysis showed that caffeine could strikingly inhibit the formation of dimeric G6PDH (Figure 2L), which was consistent with the results of SPR studies and molecular docking studies. Together, these findings further support that caffeine targets G6PDH and disrupts its enzymatic activity.

### Caffeine Suppresses RCC Cell Proliferation and G6PDH Activity *in vitro*

**FIGURE 3:**
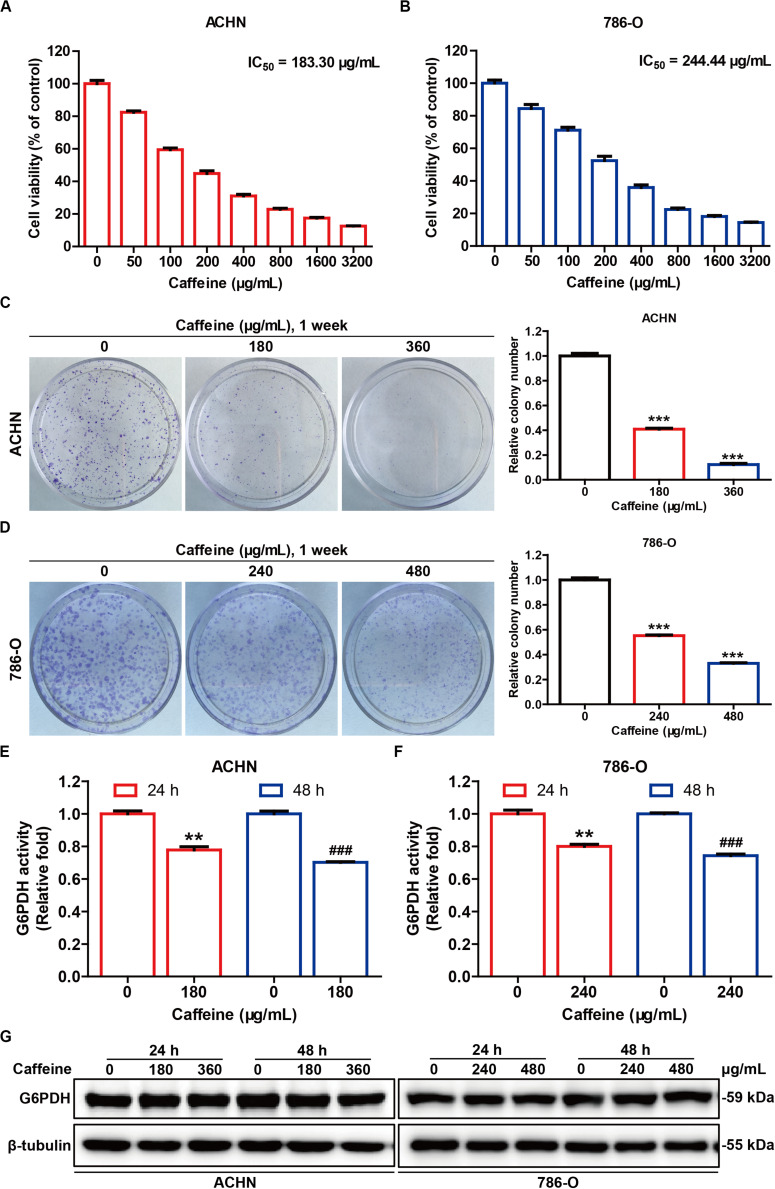
Caffeine suppresses RCC cell proliferation and G6PDH activity *in vitro*. The inhibitory effect of caffeine on ACHN **(A)** and 786-O **(B)** cell growth, as measured by MTT assay. The IC_50_ values were calculated at 48 h. Clonogenic survival assay of ACHN **(C)** and 786-O **(D)** cells treated with caffeine at the indicated concentrations. ***P < 0.001 versus the control group. G6PDH activity in ACHN **(E)** and 786-O **(F)** cells treated with caffeine at the indicated conditions, as measured using a G6PDH Activity Colorimetric Assay Kit. **P < 0.01 versus the control group at 24 h; ^###^*P* < 0.001 versus the control group at 48 h. **(G)** The expression level of G6PDH in ACHN and 786-O cells treated with caffeine, as determined by western blotting. Representative images are displayed. Data are shown as means ± SEM of triplicated experiments.

Increased G6PDH activity is closely associated with all cancers, including kidney cancer (Spencer and Stanton, 2017), and represents an important prognosticator for poor outcome in RCC, as well as a potential therapeutic target for developing effective RCC treatment strategies (Zhang et al., 2017b). To observe the effect of caffeine on G6PDH activity in a cellular system, we examined cell viability, colony formation, cell apoptosis, G6PDH activity, and G6PDH protein expression in ACHN and 786-O cells. The viability of ACHN and 786-O cells treated for 48 h with different concentrations of caffeine (from 50 to 3200 μg/mL) was determined by MTT assay. We found that caffeine inhibited the viability of both cell types in a concentration-dependent manner at the IC_50_ values of 183.30 and 244.44 μg/mL, respectively (Figures 3A,B). Based on these data, we selected the concentrations of 180 and 360 μg/mL for the ACHN cell line and 240 and 480 μg/mL for the 786-O cell line for subsequent experiments. To confirm the inhibitory effects of caffeine on cell viability, we performed clonogenicity assays (Figures 3C,D), and observed a concentration-dependent reduction of colony formation in ACHN and 786-O cells treated with caffeine. In addition, flow cytometry analysis showed that caffeine treatment also induced apoptosis in ACHN and 786-O cells (Supplementary Figure S1). As expected, caffeine treatment significantly inhibited the catalytic activity of G6PDH, whereas its protein expression was not affected in either cell line (Figures 3E–G). These results demonstrated that caffeine reduces the viability, proliferation, and G6PDH enzymatic activity of RCC cells.

### Inhibition of G6PDH Abrogates the Inhibitory Effects of Caffeine on RCC Cell Growth

**FIGURE 4:**
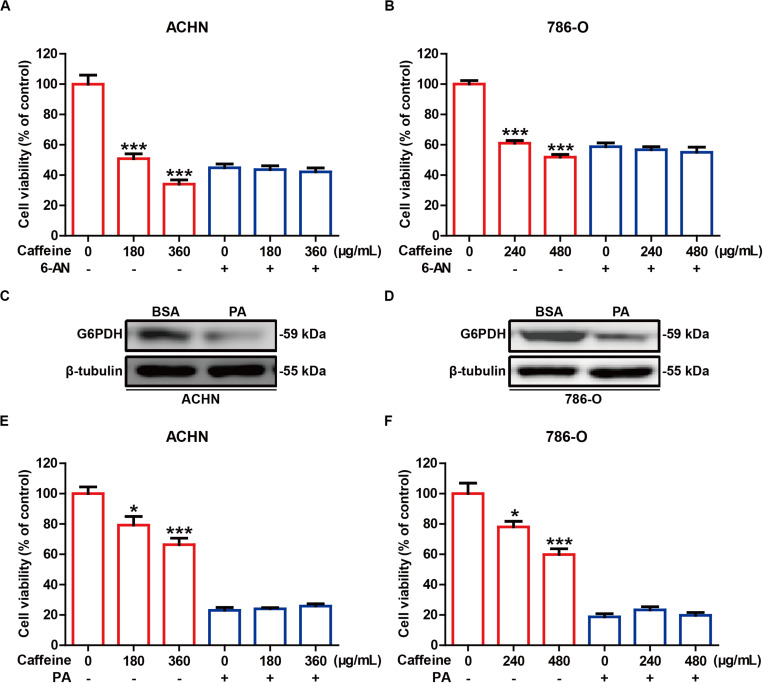
G6PDH inhibition abrogates the inhibitory effects of caffeine on RCC cell growth. **(A,B)** ACHN and 786-O cells were pretreated with or without 6-AN for 4 h and subsequently stimulated with caffeine for 48 h, and then subjected to MTT assay. ***P < 0.001 versus the control group. **(C,D)** The expression level of G6PDH in ACHN and 786-O cells treated with PA, as determined by western blotting. Representative images are displayed. **(E,F)** ACHN and 786-O cells were pretreated with or without PA for 4 h and subsequently stimulated with caffeine for 12 h, and then subjected to MTT assay. *P < 0.05 and ***P < 0.001 versus the control group. Data are shown as means ± SEM of triplicated experiments.

To investigate whether the inhibitory effects of caffeine on RCC cell growth are dependent on G6PDH, we undertook inhibition of G6PDH in RCC cells. 6-AN is a competitive biochemical inhibitor of G6PDH (Chen et al., 2018). ACHN and 786-O cells were pretreated with or without 6-AN for 4 h and subsequently stimulated with caffeine for 48 h. As expected, inhibition of G6PDH with 6-AN abrogated the inhibitory effects of caffeine on RCC cell viability (Figures 4A,B). Previous studies have shown that lipids such as PA could significantly reduce the protein expression of G6PDH, which results in similar results with G6PDH knockdown (Wang et al., 2019; Yang et al., 2019). The inhibitory effect of PA on G6PDH expression in ACHN and 786-O cells was confirmed by western blotting analysis (Figures 4C,D). To further confirm that caffeine exerts its inhibitory effects on RCC cell growth through acting on G6PDH, ACHN and 786-O cells were pretreated with or without PA for 4 h and subsequently stimulated with caffeine for 12 h. Consistently, inhibition of G6PDH with PA also abrogated the inhibitory effects of caffeine on RCC cell growth (Figures 4E,F). Collectively, these results demonstrated that caffeine didn’t result in further growth inhibitory effects in RCC cells treated with G6PDH inhibitor and that G6PDH is a major target for caffeine for inhibition of RCC development and progression.

### Caffeine Disrupts G6PDH-Mediated Redox Homeostasis in RCC Cells

**FIGURE 5:**
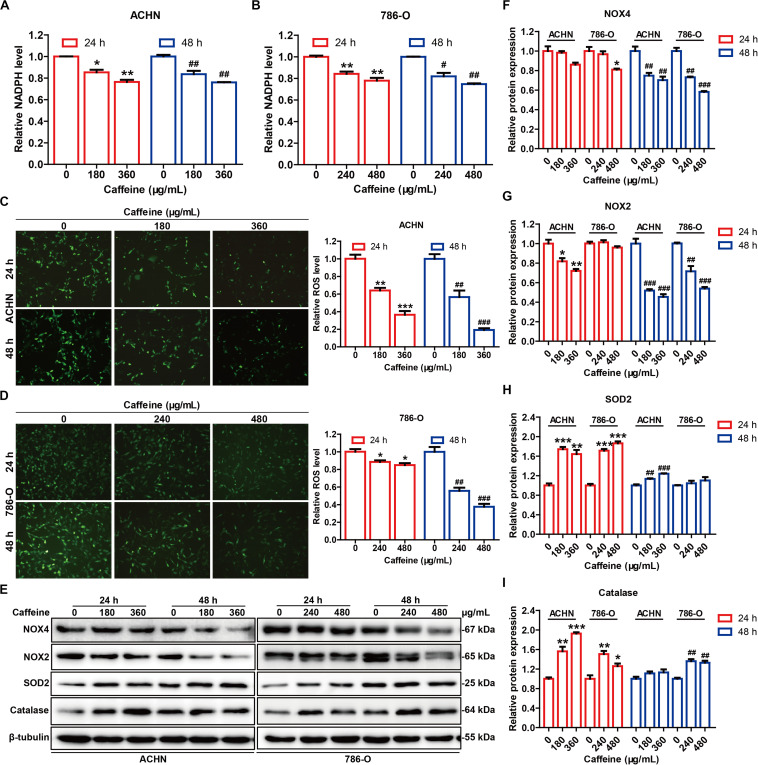
Caffeine disrupts G6PDH-mediated redox homeostasis in RCC cells. NADPH levels in ACHN **(A)** and 786-O **(B)** cells treated with caffeine, as detected using a NADP/NADPH Quantification Kit. *P < 0.05 and **P < 0.01 versus the control group at 24 h; ^#^*P* < 0.05 and ^##^*P* < 0.01 versus the control group at 48 h. Intracellular ROS accumulation in ACHN **(C)** and 786-O **(D)** cells treated with caffeine, as determined using a general oxidative stress indicator (CM-H_2_DCFDA). Images were captured at × 200 magnification. *P < 0.05, **P < 0.01, and ***P < 0.001 versus the control group at 24 h; ^##^*P* < 0.01 and ^###^*P* < 0.001 versus the control group at 48 h. **(E)** Expression levels of redox-related proteins in ACHN and 786-O cells treated with caffeine, as determined by western blotting. The gray densities of the bands corresponding to NOX4 **(F)**, NOX2 **(G)**, SOD2 **(H)**, and catalase **(I)** proteins were quantified using AlphaView software. *P < 0.05, **P < 0.01, and ***P < 0.001 versus the control group at 24 h; ^##^*P* < 0.01 and ^###^*P* < 0.001 versus the control group at 48 h. Representative images are displayed. Data are shown as means ± SEM of triplicated experiments.

G6PDH is the rate-limiting enzyme of the PPP, and acts as a guardian of cellular redox homeostasis by cooperating with NADPH oxidases (NOXs) and synergistically regulating the generation of ROS (Zhang et al., 2017a; Yang et al., 2018), which play critical roles in tumor development and progression (Liou and Storz, 2010). Given the inhibitory effect of caffeine on G6PDH activity, we speculated that caffeine could disrupt the G6PDH-regulated cellular redox homeostasis. To test this hypothesis, we measured the NADPH and ROS levels, as well as the expression levels of NOX4, NOX2, SOD2, and catalase in caffeine-treated RCC cells (Figure 5). As expected, caffeine treatment reduced the intracellular levels of NADPH (Figures 5A,B), inhibited ROS accumulation (Figures 5C,D and Supplementary Figure S2), and downregulated the expression levels of NOX4 and NOX2 (Figures 5E–G) in both ACHN and 786-O cells. Moreover, the protein expression levels of antioxidases, including SOD2 and catalase, were increased in caffeine-treated RCC cells (Figures 5E,H,I). These results demonstrated that caffeine could alter the G6PDH-regulated redox homeostasis in RCC cells.

Additionally, G6PDH is reported to promote RCC proliferation through positive feedback regulation of phosphorylated signal transducer and activator of transcription 3 (p-STAT3) via upregulated cyclin D1 expression (Zhang et al., 2017a). Here, we investigated whether caffeine could suppress the expression levels of p-STAT3 and cyclin D1 in RCC cells. Western blot analysis showed that the p-STAT3/STAT3 ratio and the protein expression level of cyclin D1 were decreased in ACHN and 786-O cells treated with caffeine (Supplementary Figure S3). However, caffeine treatment did not affect the protein expression level of STAT3 in either cell line (Supplementary Figure S3A). Collectively, the above results suggested that caffeine could block the positive feedback regulation between G6PDH and p-STAT3 in RCC cells.

### Caffeine Reduces Tumor Growth Through Inhibition of G6PDH Activity in RCC Cell-Based Xenografts

**FIGURE 6:**
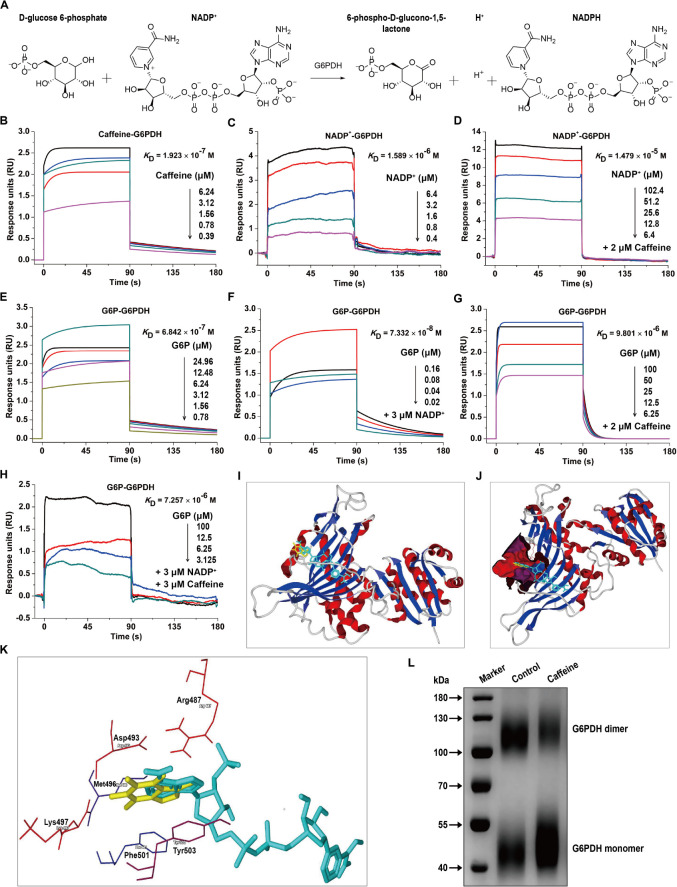
Caffeine suppresses xenografted RCC development by inhibiting G6PDH activity *in vivo*. Caffeine treatment inhibits the tumor volume and tumor weight of ACHN **(A–C)** and 786-O **(D–F)** human RCC xenografts in nude mice, but does not affect the body weight **(G)** of these tumor-bearing mice. **(H,I)** G6PDH activity in xenografted ACHN **(H)** and 786-O **(I)** tumor tissues, as measured using a G6PDH Activity Colorimetric Assay Kit. **(J)** Immunohistochemical staining of ACHN and 786-O tumor tissue sections with antibodies against Ki67, p-STAT3, and STAT3 (original magnification × 400). The expression level of Ki67 **(K)** and the p-STAT3/STAT3 ratio **(L)** were analyzed using Image-Pro Plus 6.0 software. **(M)** Western blotting analysis of the xenografted ACHN and 786-O tumor tissues with the indicated antibodies. *P < 0.05, **P < 0.01, and ***P < 0.001, low-dose caffeine group versus the control group; ^#^*P* < 0.05, ^##^*P* < 0.01, and ^###^*P* < 0.001, high-dose caffeine group versus the control group. Representative images are displayed. Data are shown as means ± SEM of five mice per group.

To further investigate whether caffeine can inhibit tumor growth through inhibition of G6PDH activity *in vivo*, ACHN and 786-O cells in the logarithmic growth phase were subcutaneously injected into the flanks of nude mice. After 2 weeks, caffeine (60 or 120 mg/kg body weight/day) was administered continuously for 34 days *via* the intragastric route. ACHN and 786-O tumor xenograft-bearing mice treated with caffeine both exhibited a significant reduction in tumor volume and tumor weight (Figures 6A–F), whereas caffeine treatment did not affect the body weight of these mice (Figure 6G). As expected, caffeine treatment significantly inhibited G6PDH activity in tumor tissues of ACHN and 786-O xenografts (Figures 6H,I). Additionally, immunohistochemical staining of the tumor sections demonstrated that caffeine treatment induced a significant decrease in the expression levels of Ki67 (a specific marker for cell proliferation) as well as in the ratio of p-STAT3/STAT3; however, caffeine treatment did not affect the protein expression of STAT3 (Figures 6J–L). Furthermore, western blotting analysis of the tumor tissues from ACHN and 786-O xenografts indicated that caffeine treatment did not affect the expression of G6PDH and STAT3; however, caffeine treatment led to the downregulation of the expression of NOX4, p47-phox, p-STAT3, and cyclin E, whereas the expression of SOD2 was upregulated (Figure 6M). Taken together, these results demonstrated that caffeine treatment can reduce tumor growth by inhibiting G6PDH activity *in vivo*, which was highly consistent with the results of the *in vitro* studies.

## Discussion

**FIGURE 7:**
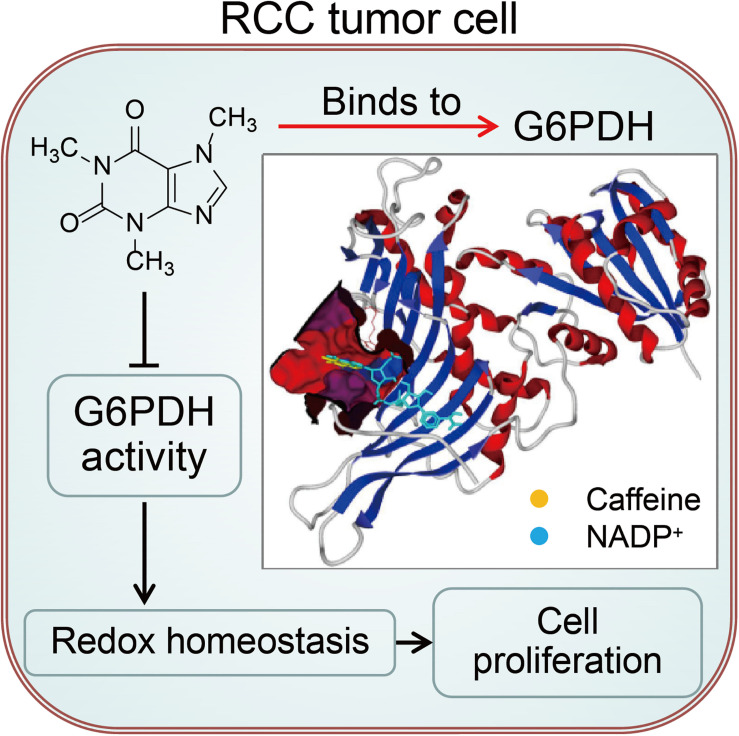
A working model for caffeine targets G6PDH to disrupt redox homeostasis and inhibit RCC cell proliferation.

Defining and then exploiting the molecular requirements that distinguish tumors from normal tissues is the hallmark of cancer research (Liberti et al., 2017). G6PDH is the rate-limiting enzyme in the PPP, a major pathway for glucose metabolism, and is considered to play oncogenic roles based on its overexpression and high enzymatic activity in various tumors (Wang et al., 2012; Zhang et al., 2014, 2017b; Spencer and Stanton, 2017). The G6PDH-regulated PPP has been understood largely in terms of its role as a source of reducing power and ribose phosphate for the cell for the maintenance of redox balance and biosynthesis of nucleotides and lipids, which are essential for cancer cell growth (Zhang et al., 2014). As a consequence, G6PDH is considered a tumor biomarker and a potential therapeutic target for cancer treatment. However, full target inhibition, and consequently complete ablation of an enzyme involved in glucose metabolism, is difficult to achieve because glucose metabolism is required in nearly every mammalian tissue (Liberti et al., 2017). As such, it is important to identify mechanisms that induce partial inhibition of metabolic enzyme activity with selectivity against tumors. In the present study, we identified a hitherto unknown biochemical mechanism involving the caffeine-mediated regulation of G6PDH activity that disrupts cellular redox homeostasis and suppresses RCC development and progression (Figure 7).

Caffeine, an important natural secondary metabolite, was first isolated in the 18th century (Huang et al., 2014). Approximately 87% of the world’s population consumes an average of 193 mg of caffeine every day. Caffeine is present in several plant leaves, fruits, barks, and seeds, where it plays important roles in protecting plants from herbivores, pathogens, and physical stresses (Verster and Koenig, 2018). Importantly, this botanically sourced natural small molecule compound (average mass: 194.191 Da) has attracted widespread interest owing to its broad-spectrum pharmacological activities across a wide range of doses (Cappelletti et al., 2015; Tej and Nayak, 2018), including its antioxidant (Li et al., 2018), antitumor (Okano et al., 2008; Edling et al., 2014), and antidiabetic activities (Fang et al., 2015), as well as its role in the regulation of lipid metabolism (Du et al., 2018b; Fang et al., 2019) and lifespan-extending properties (Du et al., 2018a). Caffeine is generally recognized as safe by the Food and Drug Administration, and is absorbed very quickly and peak plasma concentration of 15.9–18.7 μg/mL usually occurs 0.5 h after the ingestion of 500 mg of caffeine (Huang et al., 2014). The ADORA2A is well-known as the primary cellular target of caffeine, with a binding affinity of approximately 10 μM (Fredholm, 1995). In this study, SPR analysis demonstrated that caffeine could directly bind to G6PDH, the rate-limiting enzyme in the PPP, with high affinity (*K*_D_ = 0.1923 μM), and compete with the coenzyme NADP^+^ for G6PDH binding, as demonstrated by the observed reduced binding affinities of G6PDH for its coenzyme and substrate following caffeine treatment. Furthermore, caffeine didn’t result in further growth inhibitory effects in RCC cells treated with G6PDH inhibitor, suggesting that G6PDH is a major target for caffeine for inhibition of RCC development and progression.

The structure of human G6PDH indicates that each subunit contains two NADP^+^ binding sites, a catalytic NADP^+^ coenzyme-binding domain, and a structural NADP^+^ binding domain (Wang et al., 2008, 2014). Fluorescence titration of the stripped enzyme gave the *K*_D_ for structural NADP^+^ as 37 nM, 200-fold lower than for “catalytic” NADP^+^ (Wang et al., 2008). Our molecular docking studies further revealed that caffeine binds to the structural NADP^+^ binding domain of G6PDH. More importantly, chemical cross-linking analysis showed that caffeine (80 μM) could strikingly inhibit the formation of dimeric G6PDH. Based on these facts and results, it is very possible that caffeine binds to both structural NADP^+^-binding site and the catalytic center at high doses, although this needs further verification in the future. Together, these findings support that G6PDH is a cellular target for caffeine. In addition, given that caffeine, NADP^+^, and NAD^+^ all show a similar purine structure, and that the NADP^+^- or NAD^+^-dependent reactions catalyzed by metabolic enzymes are ubiquitous in living organisms, we further speculate that caffeine might also affect other NADP^+^- or NAD^+^-dependent metabolic enzymes such as the NAD^+^-dependent and rate-limiting glycolytic enzyme glyceraldehyde-3-phosphate dehydrogenase (GAPDH), which is also known to play key roles in cancer development and progression (Yun et al., 2015; Liberti et al., 2017). However, these hypotheses need further investigation, and are the subject of ongoing work in our laboratory.

As previously reported, overexpression and high enzymatic activity of G6PDH are frequently observed in various tumors, including RCC (Zhang et al., 2017b; Yang et al., 2019). In this study, we demonstrated that caffeine can inhibit G6PDH activity in a pure enzyme reaction system. Consequently, we hypothesized that caffeine might inhibit RCC cell proliferation through inhibition of G6PDH. As expected, we found that caffeine treatment significantly suppressed RCC cell proliferation in a concentration-dependent manner. We also found that, in RCC protein lysates, caffeine inhibited G6PDH enzymatic activity, but did not affect its expression. As G6PDH is the rate-limiting enzyme in the PPP, inhibition of G6PDH would be expected to affect NADPH production and lead to redox imbalance (Mele et al., 2018). Accordingly, we found that caffeine-mediated inhibition of G6PDH activity resulted in a reduction of intracellular NADPH and ROS levels. ROS act as an important second messenger in cell signaling and are essential for numerous biological processes in normal cells. Any aberrance in redox homeostasis are closely associated with human pathogenesis including cancers. Because of the double-edged sword property of ROS in determining cell fate, both pro- or anti-oxidant therapies have been proposed for treatments of cancers (Wang and Yi, 2008; Liou and Storz, 2010). Although the cellular functions of ROS in cancer remain controversial, many cancer cells show a sustained increase in the intrinsic production of ROS, which maintains the oncogenic phenotype and promotes tumor progression (Liou and Storz, 2010; Sullivan and Chandel, 2014). The sources of ROS and their activation within subcellular compartments will change over a timeline of tumor evolvement and contribute to tumor heterogeneity (Shanmugasundaram and Block, 2016). NADPH is a substrate of NOX oxidases, which are prominent ROS generators. NOX oxidases comprise seven members (NOX1 to NOX5, DUOX1, and DUOX2). NOX4, the major isoform in the kidney, is the main source of ROS in RCC (Zhang et al., 2017a; Yang et al., 2018). In the present study, we found that caffeine treatment led to the downregulation of the protein expression of NOX4 and NOX2, as well as the upregulation of antioxidant protein expression in RCC cells, including that of SOD2 and catalase, which are closely associated with the antioxidant effects of caffeine. As such, the results of the current study might provide new insights into the mechanism underlying the antioxidant effects of caffeine.

Studies have shown that G6PDH promotes tumor cell proliferation mainly *via* ROS-stimulated p-STAT3 signaling activation and upregulation of cyclin D1 expression in RCC cells (Zhang et al., 2017a). In this study, caffeine inhibited the activation of p-STAT3 signaling and downregulated the expression of the cyclin D1 protein in RCC cells, suggesting that caffeine can affect the cell cycle phase distribution of RCC cells (data not shown). Importantly, we further demonstrated that caffeine could reduce tumor growth in a RCC cell xenograft mouse model. Consistent with the *in vitro* results, caffeine significantly inhibited G6PDH enzymatic activity, p-STAT3 signaling activation, and cyclin E, NOX4, and p47-phox (NOX2 regulatory subunit) protein expression, and upregulated the expression of SOD2 protein, in xenografted RCC tumor tissues. These effects eventually led to a redox imbalance and inhibition of tumor growth. Consequently, regular consumption of caffeine in the form of tea or coffee may be a convenient and feasible method to treat RCC patients whose cells show high G6PDH activity, although the clinical application of such an approach needs further verification.

## Conclusion

Our findings revealed that caffeine can target G6PDH, thereby disrupting redox homeostasis and inhibiting RCC tumor growth. Caffeine may represent a potential therapeutic agent for the treatment of RCC.

## Data Availability Statement

All datasets generated for this study are included in the article/Supplementary Material.

## Ethics Statement

The animal study was reviewed and approved by Yunnan Agricultural University Institutional Ethics Committee.

## Author Contributions

JS, XW, and HX designed the study. HX, LH, TL, FC, JL, JX, and LJ performed the experiments. HX, LH, and TL analyzed the data. JS, XW, and ZX contributed reagents, materials, and analysis tools. HX and TL wrote and revised the manuscript. All authors read and approved the final version of the manuscript.

## Conflict of Interest

The authors declare that the research was conducted in the absence of any commercial or financial relationships that could be construed as a potential conflict of interest.

## Footnotes

1 http://www.pdb.org

## Abbreviations

6-AN: 6-aminonicotinamide
ADORA2A: adenosine A2a receptor
DMEM: Dulbecco’s modified Eagle’s medium
FBS: fetal bovine serum
G6P: D-glucose-6-phosphate
G6PDH: glucose-6-phosphate dehydrogenase
GAPDH: glyceraldehyde-3-phosphate dehydrogenase
*K*_D_: equilibrium dissociation constant
MTT: 3-(4,5-dimethylthiazol-2-yl)-2,5-diphenyltetrazolium bromide
MVD: molegro virtual docker
NADPH: nicotinamide adenine dinucleotide phosphate
NOXs: NADPH oxidases
PA: palmitate
PI: propidium iodide
PMSF: phenylmethylsulfonyl fluoride
PPP: pentose phosphate pathway
P/S: penicillin–streptomycin solution
p-STAT 3: phosphorylated signal transducer and activator of transcription 3
PVDF: polyvinylidene fluoride
RCC: renal cell carcinoma
RIPA: radioactive immunoprecipitation assay
ROS: reactive oxygen species
SEM: standard error of the mean
SPR: surface plasmon resonance.
