# Supplementary material for: Caffeine Targets G6PDH to Disrupt Redox Homeostasis and Inhibit Renal Cell Carcinoma Proliferation
Source: Front Cell Dev Biol. 2020 Oct 6;8:556162. doi: 10.3389/fcell.2020.556162 (PMC7573228; doi:10.3389/fcell.2020.556162)
Supplement: Supplementary file 1 [file Data_Sheet_1.PDF]

Supplementary Materials

Caffeine Targets G6PDH to Disrupt Redox Homeostasis and Inhibit Renal Cell Carcinoma Proliferation

Huanhuan Xu[1,2†], Lihong Hu[1,3†], Titi Liu[1,2†], Fei Chen[1,3], Jin Li[1,3], Jing Xu[1,3], Li Jiang[1,3], Zemin Xiang[1,3]*, Xuanjun Wang[1,2,4]* and Jun Sheng[1,4]*

***Correspondence to:** Key Laboratory of Pu-er Tea Science, Ministry of Education, Yunnan Agricultural University, No. 452, Fengyuan Road, Panlong District, Kunming 650201, China. E-mail addresses: xiangzmwdx@sohu.com (Zemin Xiang); xuanjunwang@qq.com (Xuanjun Wang); shengj@ynau.edu.cn (Jun Sheng).

[†]These authors have contributed equally to this work.

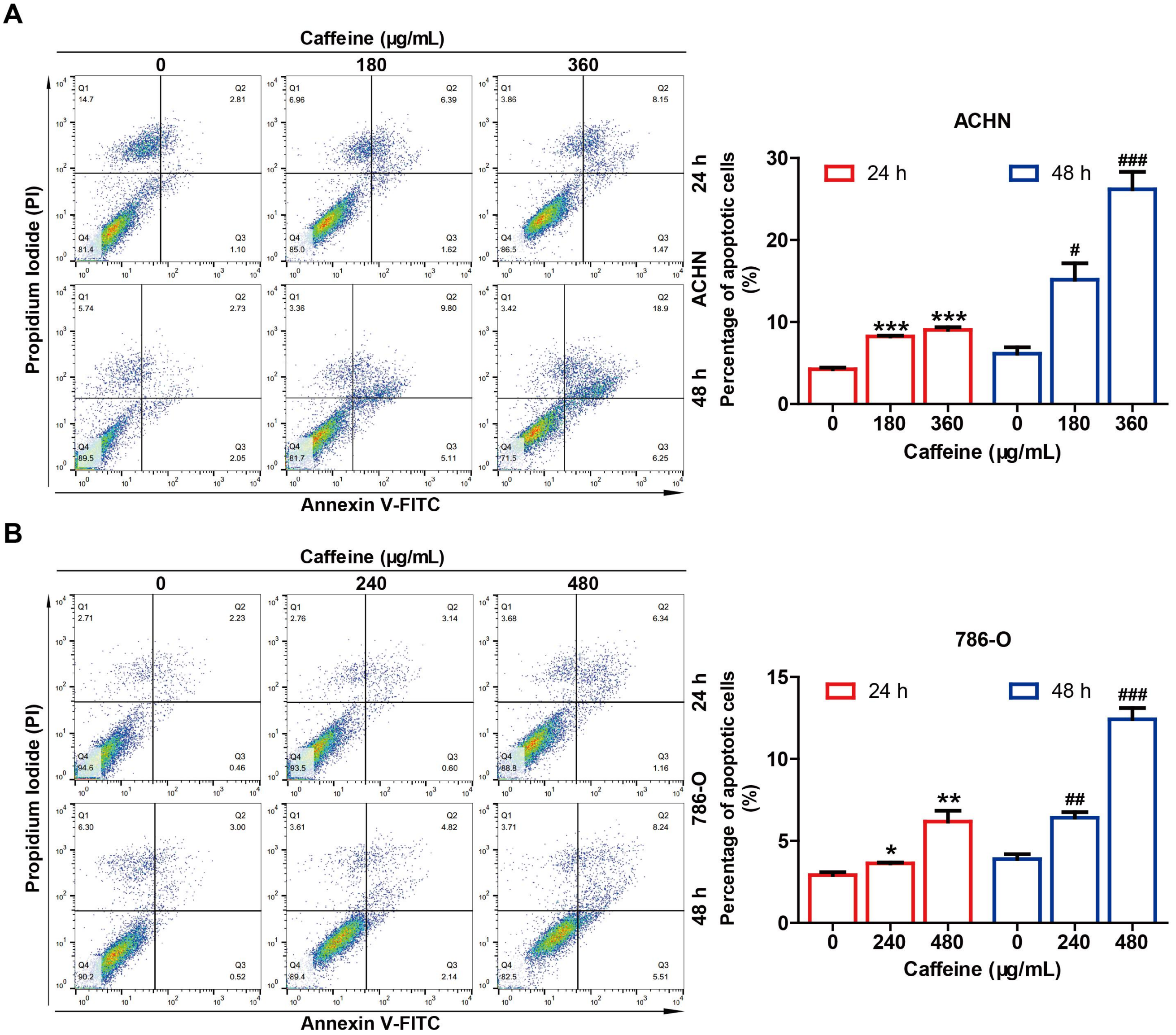

Supplementary Figure S1. Caffeine induces apoptosis in ACHN and 786-O cells. **(A,B)** Flow cytometry was used to detect cell apoptosis in ACHN and 786-O cells treated with various concentrations of caffeine. The ratio of apoptotic cells in each group are expressed as percentages. $^{*}P < 0.05$, $^{**}P < 0.01$, and $^{***}P < 0.001$ *versus* the control group at 24 h; $^{\#}P < 0.05$, $^{\#\#}P < 0.01$, and $^{\#\#\#}P < 0.001$ *versus* the control group at 48 h. Representative images are displayed. Data are shown as means ± SEM of triplicated experiments.

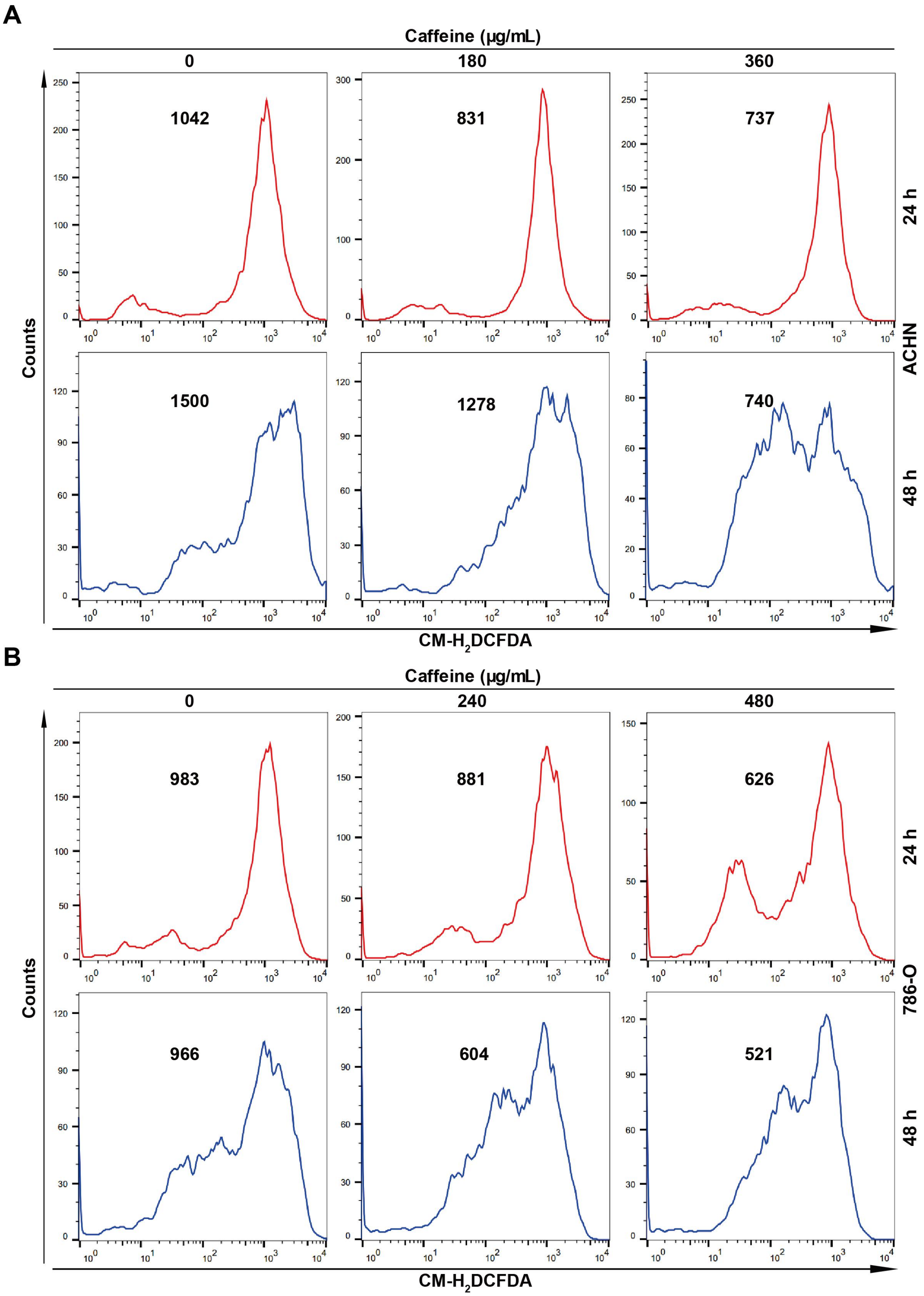

Supplementary Figure S2. Caffeine inhibits ROS production in ACHN and 786-O cells. **(A,B)** Flow cytometry was used to detect intracellular ROS levels in ACHN and 786-O cells treated with various concentrations of caffeine. The mean of fluorescence intensity is indicated and one representative experiment out of 3 is shown.

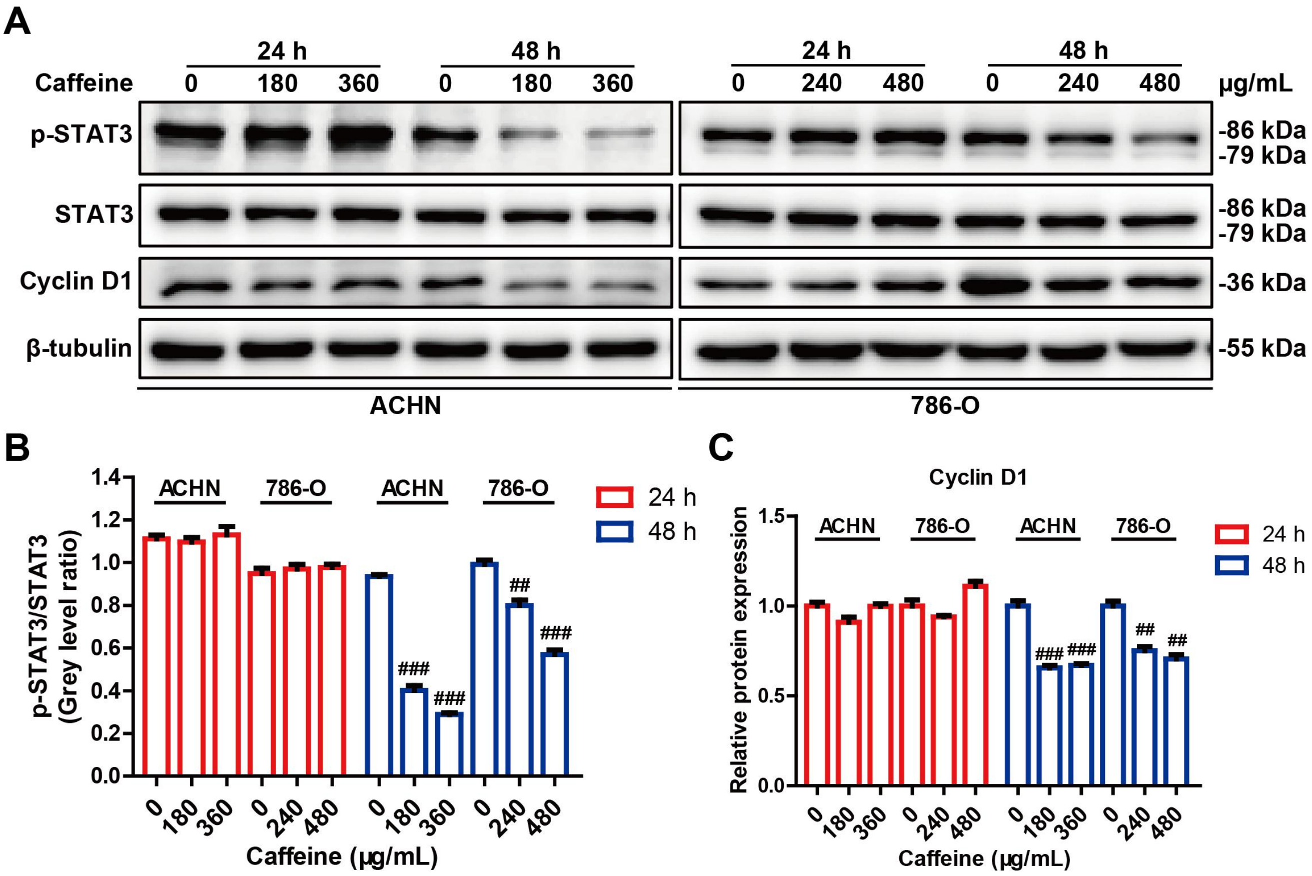

Supplementary Figure S3. Caffeine inhibits p-STAT3 signaling activation and cyclin D1 protein expression in RCC cells. **(A)** Expression levels of the indicated proteins in ACHN and 786-O cells treated with caffeine, as determined by western blotting. The ratio of p-STAT3/STAT3 **(B)** and protein expression level of cyclin D1 **(C)** were analyzed using AlphaView software. $^{##}P < 0.01$ and $^{###}P < 0.001$ *versus* the control group at 48 h. Representative images are displayed. Data are shown as means ± SEM of triplicate experiments.
